# Descriptive study of suicidal behavior in adult population attended in an emergency department during a one-year period and comparative study with the following annual period

**DOI:** 10.1192/j.eurpsy.2024.1632

**Published:** 2024-08-27

**Authors:** M. GARCÍA MORENO, A. DE COS MILAS, L. BEATOBE CARREÑO, A. IZQUIERDO DE LA PUENTE, P. DEL SOL CALDERON, R. DE ARCE CORDON

**Affiliations:** ^1^PSYCHIATRY, HOSPITAL UNIVERSITARIO PUERTA DE HIERRO MAJADAHONDA; ^2^PSYCHIATRY, HOSPITAL UNIVERSITARIO DE MÓSTOLES, MADRID, Spain

## Abstract

**Introduction:**

Suicide is the most frequent psychiatric emergency. About 1% of all deaths are due to suicide so around 700,000 people commit suicide each year. Suicide attempt is more frequent in women (3:1) while completed suicide is more frequent in men (4:1). Most suicides occur in the 35-64 age range. The severity of a suicide attempt is assessed in terms of method, potential lethality, rescuability and impulsivity. A previous suicide attempt is the main risk factor for suicide behavior. The majority (more than 90%) of suicide behavior are related to an underlying psychopathology, mainly depression and substance abuse, especially alcohol. However, there are also numerous cases of impulsive attempts in the context of life stressors.

**Objectives:**

To analyze sociodemographic and clinical characteristics of adult patients with suicidal behavior attended in the emergency department during a one-year period. To study the stability of the data obtained in the following annual period

**Methods:**

A retrospective review of the population over 18 years attended in the emergency department during 2022 because of suicidal behavior, was carried out. Data collection for the year 2023 is in progress in order to be able to carry out a comparative study between both annual periods.

**Results:**

562 patients over 18 years were attended in the emergency department of our hospital due to suicide behavior during 2022. 383 of these patients were women (68.1%) and 179 men (31.9%). with an average age of 38.6 and 42.2 years respectively. The age range between 18 and 25 years accounted for 28.5% of the total cases. The most frequent suicidal behavior was medication overdose with a total of 307 (54.6%), being more frequent in women than in men (2.6:1). The second most frequent reason for attention was suicidal ideation without suicide attempt, with a total of 212 patients (37.7%). 371 patients were discharged home from the emergency department (66%) and 191 required a longer observation in hospital environment. We are awaiting to complete data collection for 2023 to establish a comparison with those described above.

**Conclusions:**

According to our study, suicidal behavior in adult population is more frequent in women than in men. The most frequent age range in both genders was between 18 and 25 years old. The method most frequently used was medication overdose and suicidal ideation without a suicide attempt was the second most frequent reason of attention. Our patients mostly presented diagnoses of personality disorder, depression and substance use disorder.

**Disclosure of Interest:**

None Declared

